# Multiplex-PCR Detection of *Clostridium tyrobutyricum*, *Clostridium butyricum*, and *Clostridium sporogenes* in Raw Milk for Cheesemaking

**DOI:** 10.3390/life14091093

**Published:** 2024-08-30

**Authors:** Irene Floris, Francesca Martucci, Angelo Romano, Giuseppina Marello, Carmela Ligotti, Daniela Manila Bianchi

**Affiliations:** 1 SC Sicurezza Alimentare, Istituto Zooprofilattico Sperimentale del Piemonte, Liguria e Valle, d’Aosta (IZSPLV), Via Bologna 148, 10154 Turin, Italy; francesca.martucci@izsto.it (F.M.); angelo.romano@izsto.it (A.R.); giuseppina.marello@izsto.it (G.M.); cligotti@aslal.it (C.L.); manila.bianchi@izsto.it (D.M.B.); 2 Azienda Sanitaria Locale (ASL) Alessandria, Via Venezia 6, 15121 Alessandria, Italy

**Keywords:** raw milk, *Clostridium* spp., multiplex PCR

## Abstract

Late blowing defects in semi-hard and hard cheeses caused by spore-forming clostridia (e.g., *Clostridium tyrobutyricum*, *Clostridium butyricum*, *Clostridium sporogenes*) pose a major issue for the dairy industry. With this study, we applied a multiplex PCR for the rapid and simultaneous detection of clostridia in raw milk for cheese production. Spore detection in milk usually relies on culture-dependent methods, among which the most probable number (MPN) technique is sensitive but time-consuming and nonspecific. We tested two PCR-based protocols: the one entailed direct milk analysis with results obtained within 24 h; the other included an enrichment step and gave results within 72 h. The second protocol was found to be more sensitive; it detected concentrations as low as 100 cells/L for *C. sporogenes* and *C. butyricum* and 800 cells/L for *C. tyrobutyricum*. Both protocols were applied to field samples (211 samples underwent protocol no. 1; 117 samples underwent protocol no. 2) collected from four dairy processing plants in Piedmont. The prevalence of *C. butyricum* (protocol no. 1: 9.5%; protocol no. 2: 23%) was higher than either *C. sporogenes* (0%; 9.4%) or *C. tyrobutyricum* (0%; 6.8%). Protocol no. 2 detected multiple targets in eight samples, indicating that more than one microorganism was present. Our findings underscore the importance of implementing preventive measures and early detection strategies to mitigate the risk of cheese spoilage due to clostridial contamination.

## 1. Introduction

Clostridia pose technological and food safety challenges for dairy products, especially for semi-hard and hard cheese like Grana Padano [1]. Under unfavorable conditions, clostridia can undergo sporulation, which increases resistance to conventional disinfection methods [2] and raises the risk of spoilage as spores can accumulate in milk for cheesemaking [3]. During cheese ripening, the environment provides optimal conditions for the germination of bacterial spores. Temperature, pH levels, humidity, amino acids, salt concentration, and anaerobic conditions are conducive for spore formation [4]. Once germinated, the vegetative form of the bacteria exhibits active metabolism, utilizing lactose, lactic acid, and proteins to produce butyric acid, acetic acid, and gases. The production of gases, including hydrogen and carbon dioxide, can lead to swelling, a phenomenon known as late blowing in cheese, which typically occurs after several weeks or months of aging. In addition, acid production leads to rancidity and undesirable odors [5]. Furthermore, the heat stability of spores extends the risk to products also made from pasteurized milk and to powdered milk [6], not just raw milk products. The three clostridial species responsible for cheese defects are *Clostridium tyrobutyricum*, *C. butyricum*, and *C. sporogenes* [4,5,7].

Silage is a primary source of bacteria that can be either pathogenic (e.g., *Listeria monocytogenes*, *C. botulinum*, *Bacillus cereus*, shiga toxin-producing *Escherichia coli*, *Mycobacterium bovis*) or disrupt cheese production (*C. tyrobutyricum*, *C. butyricum*, *C. sporogenes*) [8]. Clostridia spores are typically present in soil and are introduced to farms through forage contaminated with soil and/or organic fertilizers. After ingestion by animals, the spores survive passage through the gastrointestinal tract and are excreted with the feces, providing a major vehicle for contaminating both the barn environment and the milk [3,7,9,10].

Previous studies have analyzed the extent of silage contamination and the effectiveness of preventive measures [3,11,12]. Bactofugation and microfiltration are the two most common methods to reduce bacterial load in milk, especially against clostridial contamination [13,14]. However, certain physical treatments are prohibited in the production of several types of Protected Designation of Origin (PDO) cheese. While other methods are active against spore germination and gas production due to the use of additives like nitrate and lysozyme, or control ripening conditions, such as pH [15], salt concentration [1], and temperature, they are not always straightforward to apply and may not consistently prevent late swelling [4]. Alternative approaches to reduce *C. tyrobutyricum* outgrowth are the use of bacteriocins produced by lactic acid bacteria [16,17,18,19,20], aromatic plant extracts [21], or bacteriophages against gas-producing bacteria [22].

Moreover, current methods for detecting spores in milk are culture-dependent, of which the most probable number (MPN) method is widely utilized [23]. The MPN method entails assessing the ability of microorganisms, in specific culture media, to ferment lactate by measuring gas production. Typically, several days are needed to confirm a positive result and this method can be challenging in terms of specificity [24]. Rapid and automated methods for spore detection are also available commercially but necessitate specific equipment [25].

Numerous studies are underway to explore various methods for detecting clostridia [5], including real time PCR [26], end point PCR [27], denaturing gradient gel electrophoresis (DGGE) [28], PCR-temporal temperature gradient gel electrophoresis (PCR-TTGE) [29], point of care detection [30], automated ribosomal intergenic spacer analysis (ARISA) [31], and Raman spectroscopy [32]. A major disadvantage of molecular analysis is the difficulty in extracting clostridia DNA due to the spore’s thick resistant wall. Therefore, various extraction methods are being investigated [33,34].

*C. tyrobutyricum* is the predominant cause of late blowing defect in cheese. Extensive research into its phenotypic [35] and genotypic characteristics has been conducted [30,34,36,37]. A synergistic effect has been found between *C. tyrobutyricum* and other clostridial species (e.g., *C. sporogenes* and *C. butyricum*), so it follows that methods that can detect multiple species should be employed to gain a better understanding of this synergy.

Information about the prevalence of the genus *Clostridium* in silage is abundant, while data for milk and cheese are scarce. Most studies to date have used conventional methods in the analysis of cheese matrices. Conventional techniques, and the MPN method in particular, have high sensitivity but are not species specific and require up to 10 days for bacterial growth, making them impractical in cheesemaking due to the lability of the raw milk material.

With this study, we applied a multiplex PCR method that can simultaneously and quickly detect *C. tyrobutyricum*, *C. butyricum*, and *C. sporogenes* in raw milk for the production of semi-hard and hard cheese. Two analytical protocols were utilized: one entailed direct analysis of milk and yielded results within 24 h, while the other included an enrichment step and returned results within 72 h.

## 2. Materials and Methods

### 2.1. Study Design and Sampling

Protocol no. 1 entailed direct analysis of raw milk samples and Protocol no. 2 included an enrichment step to enhance method sensitivity. Both protocols utilized identical procedures for DNA extraction and amplification. To determine protocol performance, a spiking test was conducted using American Type Culture Collection (ATCC) certified strains *C. tyrobutyricum* (ATCC^®^ 25755), *C. butyricum* (ATCC^®^ 19398), and *C. sporogenes* (ATCC^®^ 11437) stored at −20 °C according to the manufacturer’s guidelines. Both protocols were applied to field samples collected from four dairy processing plants in Piedmont, which collect milk from various dairy farms. Sampling was conducted in two periods: 211 raw milk samples were collected between September and October 2021 and analyzed directly, while 117 raw milk samples were collected from June to July 2023 and analyzed after an enrichment step (Table 1). Sampling was conducted on a voluntary basis, with the number of samples depending on the dairy plant’s availability. All the dairy plants are located in the province of Cuneo in Piedmont. Plants A and D collect milk from dairy farms located in the provinces of Turin and Cuneo, while Plants B and C source their milk exclusively from farms in the province of Cuneo and are part of the Grana Padano Protection Consortium. All were informed about the study design, the nature of the data being collected, and their intended future use.

### 2.2. Strain Growth Condition and Spiking Tests

Certified bacterial strains were cultured on Columbia blood agar (Biolife, Milan, Italy) under anaerobic conditions at 37 °C for 24 h. An anaerobic environment was achieved using jars and AnaeroGen bags (Thermofisher Scientific, Waltham, MA, USA). A spiking solution was prepared from a 0.5 McFarland bacterial suspension, measured with a McFarland Densitometer (Biosan, Riga, Latvia), and serial dilutions were made. For Protocol 1, ultra-high temperature (UHT) milk was spiked separately with the three certified strains (*C. tyrobutyricum*, *C. butyricum*, *C. sporogenes*) at concentrations from 10^10^ to 10^4^ cells/mL. For Protocol 2, UHT milk was spiked with concentrations of 800 cells/L, 500 cells/L, and 100 cells/L for *C. tyrobutyricum*, *C. butyricum*, and *C. sporogenes*, separately. These concentrations were chosen to evaluate the performance of the enrichment step in enhancing sensitivity.

### 2.3. Enrichment of Milk Samples

Enrichment broths were compared to identify the most effective for detecting clostridia. The four broths evaluated were thioglycolate (THIO, Biolife), tryptone peptone glucose yeast extract (TPGYT), brain heart infusion (BHI), and reinforced clostridial medium (RCM). In detail, 50 mL of milk were centrifuged at 4200× *g* for 45 min at 4 °C. The superficial fat layer and supernatant milk were removed, and the pellet was resuspended in 10 mL of the culture medium. The samples were incubated at 37 °C under anaerobic conditions for 24, 48, and 72 h. The vials for the THIO culture medium were prepared according to the supplier’s instructions: they were heated in boiling water for 5 min with the tops partially unscrewed, then cooled to room temperature to ensure complete reduction of the medium. This comparative approach served to determine which enrichment broth provided the best conditions for growth and detection of the bacteria in the milk samples.

### 2.4. DNA Extraction and Purification

A MagMAX™ CORE Mastitis and Panbacteria Module Kit (Thermofisher Scientific) was used for extraction; the protocol was provided by the manufacturer for milk samples. In detail, 25 mL of raw milk were centrifugated at 4200× *g* for 45 min at 4 °C. The superficial fat was removed and only 2 mL of supernatant was left. The pellet was resuspended in the 2 mL of supernatant and transferred into a MagMAX CORE bead beating tube. The culture broths were centrifuged at 8000× *g* for 15 min at 4 °C and the pellet was resuspended in 2 mL of demineralized water and transferred into a MagMAX CORE bead beating tube. The subsequent steps were identical for both protocols. The samples were centrifuged at 15,000× *g* for 10 min and the supernatant was removed. The pellet was resuspended in 400 μL of MagMAX CORE clarifying solution and then put in a Disruptor Genie for 25 min to disrupt the cells. After centrifugation at 15,000× *g* for 3 min, 300 μL of the supernatant lysate was transferred to a new clean tube and incubated with 10 μL of proteinase K for 2 min at room temperature. 

A mixture was prepared for DNA purification of each sample: 350 μL di MagMAX CORE lysis solution, 350 μL di MagMAX CORE binding solution, and 20 μL di MagMAX CORE magnetic bead. In the following step, 720 μL of the mixture was added to all samples and mixed in a vortex shaker for 10 min. The samples were then transferred to a magnetic stand for 1 min and the supernatant was removed. For the first wash, 500 μL of Mag MAX CORE wash solution 1 was added to each sample and vortexed for 1 min. After 1 min in the magnetic stand, the supernatant was removed. The second wash with Mag MAX CORE wash solution 2 proceeded in same way. After removing the supernatant, the samples were left open for 5 min to dry the beads. For the elution step, 90 μL of Mag MAX CORE elution buffer was added to the beads and vortexed for 10 min. The samples were then incubated for 2 min in the magnetic stand and the supernatant was stored at −20 °C until analysis.

### 2.5. Amplification

The amplification protocol and primers in this study were developed by Cremonesi et al. [27] (Table 2). The thermal profile was optimized for the Taq QIAGEN Multiplex PCR Kit (Qiagen, Hilden, Germany). Each PCR reaction was carried out in a total volume of 25 μL, consisting of 10 μL of Master Mix (QIAGEN Multiplex PCR Kit), 1.5 μL of primer Cl-SPOR-F3031, 1.5 μL of primer Cl-SPOR-R3579, 0.75 μL of primer Cl-BUTY-F1329, 0.75 μL of primer Cl-BUTY-R1640, 0.75 μL of primer Cl-TYRO-F1253, 0.75 μL of primer Cl-TYRO-R1462, 4 μL of Q solution (QIAGEN Multiplex PCR Kit), and 5 μL of DNA template.

Reactions were carried out on an ABIPRISM thermocycler (Thermofisher Scientific) as follows: initial denaturation at 95 °C for 5 min, then 30 cycles at 95 °C for 1 min, at 56 °C for 1 min, and at 72 °C for 1 min. Final extension at 72 °C for 5 min. 

The amplicons were analyzed by capillary electrophoresis with a QIAxcel DNA Screening kit on a QIAxcel Advanced System (QIAGEN) instrument. The AM420 method (injection time 10 s) was applied.

## 3. Results

### 3.1. Spiked Samples

Under Protocol 1, DNA from *C. sporogenes* and *C. butyricum* were detectable at concentrations of up to 10^5^ cells/L and the DNA from *C. tyrobutyricum* was detected at a concentration of 10^10^ cells/L. To improve sensitivity, the samples with a lower contamination underwent Protocol 2. Since no bacterial growth was observed in the THIO, RCA, and BHI culture broths, no further tests were performed. In the TPGYT-enriched samples, DNA amplification was observed at concentrations of up to 100 cells/L for *C. sporogenes* and *C. butyricum*, and 800 cells/L for *C. tyrobutyricum*. Subsequent analysis of field samples was carried out using the TPGYT culture broth.

### 3.2. Dairy Plant Samples

In total, 20 of the 211 samples analyzed under Protocol 1 tested positive for target DNA from *C. butyricum* (9.5%). None tested positive for *C. sporogenes* or *C. tyrobutyricum*. Positivity rates for the four processing plants were: 6.93% for Dairy plant A, 11.11% for Dairy plant C, and 14.55% for Dairy plant D; none of the samples from Dairy plant B tested positive for any of the targets.

**Figure 1:**
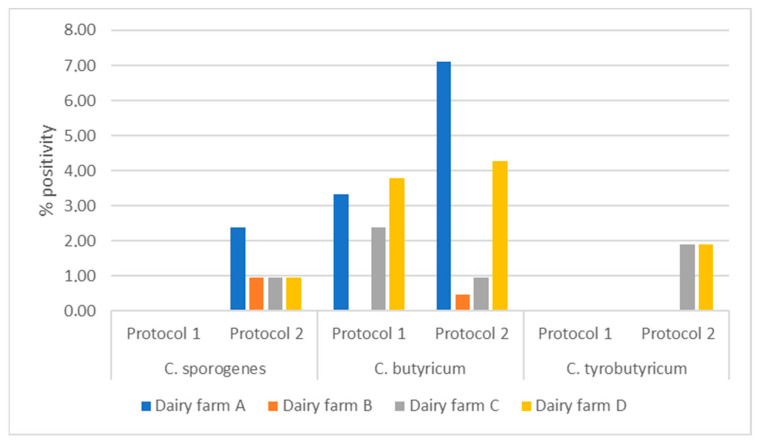
Positivity Rate of the Samples from the Four Dairy Plants for the Two Sampling Periods and Analyzed under the Two Protocols.

In total, 37 (31.6%) of the 117 samples analyzed under Protocol 2 tested positive: 27 for *C. butyricum* (23%), 11 for *C. sporogenes* (9.4%), and 8 for *C. tyrobutyricum* (6.8%). Table 3 presents the positivity rate each dairy plant. Figure 1 illustrates the positivity rates obtained with the two protocols.

A total of 8 samples were found to be positive for the target genes of more than one microorganism. Among these, one sample tested positive for all three targets (*C. tyrobutyricum*, *C. butyricum*, *C. sporogenes*), two tested positive for both *C. sporogenes* and *C. tyrobutyricum*, and four tested positive for both *C. butyricum* and *C. sporogenes*.

## 4. Discussion

Late blowing defects caused by clostridia are a major issue in the production of semi-hard and hard cheeses. Previous studies have investigated the prevalence of clostridia in silage [8,9,11], while their presence in cheese and milk has remained understudied [16,28]. Conventional microbiological analysis, based primarily on the MPN method, can take up to seven days and is not species specific. Multiplex PCR, meanwhile, can provide rapid results and differentiate between different bacterial species. The MPN method lacks specificity because there are no fully selective culture media for the genus *Clostridium*. As reported by Cremonesi et al. [27], multiplex PCR can specifically amplify the DNA of the targeted species.

In this study, two groups of samples were analyzed using two different protocols (Protocol 1 and Protocol 2), which were not applied concurrently because Protocol 2 was developed after Protocol 1. Since Protocol 2 demonstrated significantly better sensitivity performance, only this was used in the second round of sampling. The comparison between the two protocols is therefore purely statistical, rather than based on simultaneous application. This limitation could affect interpretation of the results from the two protocols.

Our data show a total positive rate of 9.5% under Protocol 1 and 31.6% under Protocol 2. Both protocols detected a higher prevalence of *C. butyricum* (9.5% under Protocol 1; 23% under Protocol 2) than *C. sporogenes* (0%; 9.4%) and *C. tyrobutyricum* (0%; 6.8%). Previous studies identified *C. tyrobutyricum* as the most frequent contaminating species [3,38], even in cheese without visible signs of late blowing defect [10,25,39,40]. Conversely, data from a Spanish study indicated a higher positivity for *C. sporogenes* (78.0%) than either *C. tyrobutyricum* (9.0%) or *C. butyricum* (1.8%) [16]. The heterogeneity of the data obtained from different working groups may result from differences in methods and matrices. Furthermore, several studies have shown that seasonality can also affect the presence of clostridia in both silage [9] and milk samples [10,31].

*C. tyrobutyricum* is the main cause of organoleptic defects in cheese. Research has indicated that other *Clostridium* strains, such as *C. sporogenes* and *C. butyricum*, can exacerbate these defects through synergistic interactions [4,16,29]. Few epidemiological studies have investigated this problem to date. However, in our study, eight raw milk samples tested positive for the DNA from more than one *Clostridium* species, with one sample containing DNA from all three species. Another study conducted in Italy reported positivity for *C. butyricum* and *C. tyrobutyricum* in a single sample [28]. Furthermore, Garde et al. [16] reported the isolation of more than one bacterial species in 40% of samples: *C. sporogenes* and *C. tyrobutyricum* in 18%, *C. sporogenes* and *C. beijerinckii* in 11%, *C. sporogenes* and *C. butyricum* in 4%, and *C. sporogenes*, *C. tyrobutyricum*, and *C. beijerinckii* in 7%.

The primary challenge of the present study was to develop an easy-to-implement and standardizable DNA extraction method. This was particularly difficult due to the complexity of analyzing clostridial spores. Therefore, one of the limitations is the detection limit of the method, especially that of Protocol 1: 10^2^ cells/mL for *C. sporogenes* and *C. butyricum*, and 10^7^ cells/mL for *C. tyrobutyricum*. Such rates are incompatible with early detection, which is crucial for preventing clostridial contamination before the cheesemaking process begins. The option to include an enrichment step in the protocol improved sensitivity but extended the processing time to 72 h, which is shorter than that of conventional methods yet still sufficient to provide cheesemakers with the option of allocating the milk to a different use. With the enrichment step, the detection limit was decreased to 100 cells/L for *C. sporogenes* and *C. butyricum* and 800 cells/L for *C. tyrobutyricum*. Similar results are shared by other studies, where cheese was analyzed using DGGE before and after the incubation phase [28].

## 5. Conclusions

Data on the occurrence of Clostridia appear to be limited, with little recent research available in Italy or elsewhere. An aim of this study was to update the data on the occurrence of *C. tyrobutyricum*, *C. butyricum*, and *C. sporogenes* in milk from dairy plants that source milk from various dairy farms in Piedmont for the production of semi-hard and hard cheeses. 

The data underscore the need for preventive measures and early spore detection to prevent cheese defects due to abnormal fermentation. Using the multiplex PCR method optimized in this study, we were able to detect *C. tyrobutyricum*, *C. butyricum*, and *C. sporogenes* directly from the milk matrix before processing or ripening. This method is valuable for allocating milk to differentiated production, thereby preventing defects in cheeses requiring long maturation. 

In contrast to the MPN method, which does not identify the bacterial species, the ability to simultaneously detect multiple *Clostridium* species offers the additional advantage of reducing both the cost and the time for microbiological analysis. Direct sampling from milk provided results in less than 24 h only for samples with high spore concentrations. Sensitivity was considerably higher with the 72 h enrichment step, which would make the method a better option for the dairy industry, where it is crucial to eliminate production milk with low spore concentrations. With this method, milk can be suitably allocated to other production processes, thus preventing spoilage in hard cheeses.

## Figures and Tables

**Table 1:** Distribution of Samples by Dairy Plant and Analytical Protocol.

Dairy Plant	Protocol 1	Protocol 2
A	101	50
B	10	11
C	45	16
D	55	40
total	211	117

**Table 2:** Primer Sequences and Expected Amplicon Size [24].

Primers	Gene Target	Primer Sequence (5′-3′)	Amplification Length (bp)
Cl-SPOR-F3031	*colA*	TTGGGATTTTGGGGATAACA	549
Cl-SPOR-R3579	*colA*	TCCGTATCGTTGTCGTCTTG	
Cl-BUTY-F1329	*hydA*	ATGGGTTAGGCAAGCAGAAA	321
Cl-BUTY-R1640	*hydA*	GCTGGATCTGCCTTCTCATC	
Cl-TYRO-F1253	*enr*	TGGTGTTCCACAAGAAGCTG	210
Cl-TYRO-R1462	*enr*	GCAGCTGGATTTACTGCACA	

**Table 3:** Samples Analyzed under Protocol 2 and Stratified by Dairy Plant.

Dairy Plant	Samples—No.	Samples Positive for *C. sporogenes*—No. (%)	Samples Positive for *C. butyricum*—No. (%)	Samples Positive for *C. tyrobutyricum*—No. (%)
Dairy plant A	16	2 (12.5)	2 (12.5)	4 (25)
Dairy plant B	11	2 (18.2)	1 (9)	/
Dairy plant C	50	5 (10)	15 (30)	/
Dairy plant D	40	2 (5)	9 (22.5)	4 (10)
TOTAL	117	11 (9.4)	27 (23)	8 (6.8)

## Data Availability

The raw data supporting the conclusions of this article will be made available by the authors on request.

## References

[1] Carminati D., Bonvini B., Francolino S., Ghiglietti R., Locci F., Tidona F., Mariut M., Abeni F., Zago M., Giraffa G. (2023). Low-Level Clostridial Spores’ Milk to Limit the Onset of Late Blowing Defect in Lysozyme-Free, Grana-Type Cheese. Foods.

[2] Chojecka A. (2022). Susceptibility of *Clostridium sporogenes* Spores to Selected Reference Substances and Disinfectants. Pol. J. Microbiol..

[3] Borreani G., Ferrero F., Nucera D., Casale M., Piano S., Tabacco E. (2019). Dairy Farm Management Practices and the Risk of Contamination of Tank Milk from *Clostridium* spp. and *Paenibacillus* spp. Spores in Silage, Total Mixed Ration, Dairy Cow Feces, and Raw Milk. J. Dairy Sci..

[4] Gómez-Torres N., Garde S., Peirotén Á., Ávila M. (2015). Impact of *Clostridium* spp. on Cheese Characteristics: Microbiology, Color, Formation of Volatile Compounds and off-Flavors. Food Control.

[5] Brändle J., Domig K.J., Kneifel W. (2016). Relevance and Analysis of Butyric Acid Producing Clostridia in Milk and Cheese. Food Control.

[6] Porcellato D., Kristiansen H., Finton M.D., Leanti La Rosa S., da Silva Duarte V., Skeie S.B. (2023). Composition and Fate of Heat-Resistant Anaerobic Spore-Formers in the Milk Powder Production Line. Int. J. Food Microbiol..

[7] Doyle C.J., Gleeson D., Jordan K., Beresford T.P., Ross R.P., Fitzgerald G.F., Cotter P.D. (2015). Anaerobic Sporeformers and Their Significance with Respect to Milk and Dairy Products. Int. J. Food Microbiol..

[8] Driehuis F., Wilkinson J.M., Jiang Y., Ogunade I., Adesogan A.T. (2018). Silage Review: Animal and Human Health Risks from Silage. J. Dairy Sci..

[9] Abeni F., Marino R., Petrera F., Segati G., Galli A., Carminati D. (2021). Farm Silage Facilities and Their Management for the Prevention of Anaerobic Bacteria Spore Contamination in Raw Milk. Dairy.

[10] Bermúdez J., González M.J., Olivera J.A., Burgueño J.A., Juliano P., Fox E.M., Reginensi S.M. (2016). Seasonal Occurrence and Molecular Diversity of Clostridia Species Spores along Cheesemaking Streams of 5 Commercial Dairy Plants. J. Dairy Sci..

[11] Goldsztejn M., Grenda T., Kozieł N., Sapała M., Mazur M., Sieradzki Z., Król B., Kwiatek K. (2020). Potential Determinants of *Clostridium* spp. Occurrence in Polish Silage. J. Vet. Res..

[12] Mosconi M., Fontana A., Daza M.V.B., Bassi D., Gallo A. (2023). *Clostridium tyrobutyricum* Occurrence in Silages and Cattle Feed: Use of Molecular and Simulation Data to Optimize Predictive Models. Front. Microbiol..

[13] Qian C., Martin N.H., Wiedmann M., Trmčić A. (2022). Development of a Risk Assessment Model to Predict the Occurrence of Late Blowing Defect in Gouda Cheese and Evaluate Potential Intervention Strategies. J. Dairy Sci..

[14] Fernández García L., Álvarez Blanco S., Riera Rodríguez F.A. (2013). Microfiltration Applied to Dairy Streams: Removal of Bacteria. J. Sci. Food Agric..

[15] Silvetti T., Morandi S., Brasca M. (2018). Growth Factors Affecting Gas Production and Reduction Potential of Vegetative Cell and Spore Inocula of Dairy-Related *Clostridium* Species. LWT.

[16] Garde S., Arias R., Gaya P., Nuñez M. (2011). Occurrence of *Clostridium* spp. in Ovine Milk and Manchego Cheese with Late Blowing Defect: Identification and Characterization of Isolates. Int. Dairy J..

[17] Carmen Martínez-Cuesta M., Bengoechea J., Bustos I., Rodríguez B., Requena T., Peláez C. (2010). Control of Late Blowing in Cheese by Adding Lacticin 3147-Producing Lactococcus Lactis IFPL 3593 to the Starter. Int. Dairy J..

[18] Rilla N. (2003). Inhibition of *Clostridium tyrobutyricum* in Vidiago Cheese by *Lactococcus lactis* ssp. *lactis* IPLA 729, a Nisin Z Producer. Int. J. Food Microbiol..

[19] Hassan H., St-Gelais D., Gomaa A., Fliss I. (2021). Impact of Nisin and Nisin-Producing *Lactococcus lactis* ssp. *lactis* on *Clostridium tyrobutyricum* and Bacterial Ecosystem of Cheese Matrices. Foods.

[20] Anastasiou R., Aktypis A., Georgalaki M., Papadelli M., De Vuyst L., Tsakalidou E. (2009). Inhibition of *Clostridium tyrobutyricum* by Streptococcus Macedonicus ACA-DC 198 under Conditions Mimicking Kasseri Cheese Production and Ripening. Int. Dairy J..

[21] Ávila M., Calzada J., Muñoz-Tébar N., Sánchez C., Ortiz de Elguea-Culebras G., Carmona M., Molina A., Berruga M.I., Garde S. (2023). Inhibitory Activity of Aromatic Plant Extracts against Dairy-Related *Clostridium* Species and Their Use to Prevent the Late Blowing Defect of Cheese. Food Microbiol..

[22] Ávila M., Sánchez C., Calzada J., Mayer M.J., Berruga M.I., López-Díaz T.M., Narbad A., Garde S. (2023). Isolation and Characterization of New Bacteriophages Active against *Clostridium tyrobutyricum* and Their Role in Preventing the Late Blowing Defect of Cheese. Food Res. Int..

[23] Peruzy M.F., Blaiotta G., Aponte M., De Sena M., Murru N. (2022). Late Blowing Defect in Grottone Cheese: Detection of Clostridia and Control Strategies. Ital. J. Food Saf..

[24] Brändle J., Fraberger V., Schuller K., Zitz U., Kneifel W., Domig K.J. (2017). A Critical Assessment of Four Most Probable Number Procedures for Routine Enumeration of Cheese-Damaging Clostridia in Milk. Int. Dairy J..

[25] Burtscher J., Rudavsky T., Zitz U., Domig K.J. (2024). Specificity of the AMP-6000 Method for Enumerating *Clostridium* Endospores in Milk. Foods.

[26] Morandi S., Cremonesi P., Silvetti T., Castiglioni B., Brasca M. (2015). Development of a Triplex Real-Time PCR Assay for the Simultaneous Detection of *Clostridium beijerinckii*, *Clostridium sporogenes* and *Clostridium tyrobutyricum* in Milk. Anaerobe.

[27] Cremonesi P., Vanoni L., Silvetti T., Morandi S., Brasca M. (2012). Identification of *Clostridium beijerinckii*, *Cl. butyricum*, *Cl. sporogenes*, *Cl. tyrobutyricum* Isolated from Silage, Raw Milk and Hard Cheese by a Multiplex PCR Assay. J. Dairy Res..

[28] Cocolin L., Innocente N., Biasutti M., Comi G. (2004). The Late Blowing in Cheese: A New Molecular Approach Based on PCR and DGGE to Study the Microbial Ecology of the Alteration Process. Int. J. Food Microbiol..

[29] Le Bourhis A.-G., Doré J., Carlier J.-P., Chamba J.-F., Popoff M.-R., Tholozan J.-L. (2007). Contribution of C. Beijerinckii and C. Sporogenes in Association with C. Tyrobutyricum to the Butyric Fermentation in Emmental Type Cheese. Int. J. Food Microbiol..

[30] Cecere P., Gatto F., Cortimiglia C., Bassi D., Lucchini F., Cocconcelli P.S., Pompa P.P. (2021). Colorimetric Point-of-Care Detection of *Clostridium tyrobutyricum* Spores in Milk Samples. Biosensors.

[31] Feligini M., Brambati E., Panelli S., Ghitti M., Sacchi R., Capelli E., Bonacina C. (2014). One-Year Investigation of *Clostridium* spp. Occurrence in Raw Milk and Curd of Grana Padano Cheese by the Automated Ribosomal Intergenic Spacer Analysis. Food Control.

[32] Barbiero D., Melison F., Cocola L., Fedel M., Andrighetto C., Dea P.D., Poletto L. (2024). Raman Spectroscopy Applied to Early Detection of *Clostridium* Infection in Milk. Appl. Spectrosc..

[33] Cremonesi P., Castiglioni B., Malferrari G., Biunno I., Vimercati C., Moroni P., Morandi S., Luzzana M. (2006). Technical Note: Improved Method for Rapid DNA Extraction of Mastitis Pathogens Directly from Milk. J. Dairy Sci..

[34] Esteban M., Marcos P., Horna C., Galan-Malo P., Mata L., Pérez M.D., Calvo M., Sánchez L. (2020). Evaluation of Methods for DNA Extraction from *Clostridium tyrobutyricum* Spores and Its Detection by qPCR. J. Microbiol. Methods.

[35] Burtscher J., Küller F., Dreier M., Arias-Roth E., Drissner D., Domig K.J. (2020). Characterization of *Clostridium tyrobutyricum* Strains Using Three Different Typing Techniques. Microorganisms.

[36] Podrzaj L., Burtscher J., Domig K.J. (2022). Comparative Genomics Provides Insights Into Genetic Diversity of *Clostridium tyrobutyricum* and Potential Implications for Late Blowing Defects in Cheese. Front. Microbiol..

[37] Bassi D., Fontana C., Zucchelli S., Gazzola S., Cocconcelli P.S. (2013). TaqMan Real Time-Quantitative PCR Targeting the Phosphotransacetylase Gene for *Clostridium tyrobutyricum* Quantification in Animal Feed, Faeces, Milk and Cheese. Int. Dairy J..

[38] Finton M., Skeie S.B., Aspholm M.E., Franklin-Alming F.V., Mekonnen Y.B., Kristiansen H., Porcellato D. (2024). Two-Year Investigation of Spore-Formers through the Production Chain at Two Cheese Plants in Norway. Food Res. Int..

[39] Brändle J., Fraberger V., Berta J., Puglisi E., Jami M., Kneifel W., Domig K.J. (2018). Butyric Acid Producing Clostridia in Cheese—Towards the Completion of Knowledge by Means of an Amalgamate of Methodologies. Int. Dairy J..

[40] Reindl A., Dzieciol M., Hein I., Wagner M., Zangerl P. (2014). Enumeration of Clostridia in Goat Milk Using an Optimized Membrane Filtration Technique. J. Dairy Sci..

